# Effect of a Ceramide NP-Containing Moisturizer on Skin Barrier Function in Adults with Atopic Dermatitis: A Randomized, Double-Blind, Placebo-Controlled Study

**DOI:** 10.1159/spp/aetag011

**Published:** 2026-10-13

**Authors:** Chao-ying Gu, Jia-yi Ying, Hui-bin Yin, Chun-yun Huang, Lei-hong Xiang

**Affiliations:** Department of Dermatology, Huashan Hospital, Fudan University, Shanghai, China

**Keywords:** Ceramide NP, Atopic dermatitis, Skin barrier function, Moisturizer, Randomized controlled trial

## Abstract

**Introduction:**

Atopic dermatitis (AD) is a chronic inflammatory skin disease associated with epidermal barrier dysfunction. This study aimed to evaluate the effects of a ceramide NP-containing soothing and moisturizing emollient cream on skin barrier function in adult patients with AD.

**Methods:**

In this single-center, randomized, double-blind, placebo-controlled parallel trial, 60 adults with moderate atopic dermatitis were randomly assigned (1:1) to receive standard therapy with either the soothing and moisturizing emollient cream or placebo. Skin barrier parameters and clinical outcomes were assessed on days 21 and 42.

**Results:**

Both groups demonstrated overall improvements in skin barrier parameters, including stratum corneum hydration, transepidermal water loss, and casual sebum levels, with generally more favorable trends observed in the intervention group. However, between-group differences in changes from baseline did not reach statistical significance, and no significant between-group differences were observed in skin surface pH. All clinical outcomes, including EASI, SCORAD, DLQI, and VAS scores for pruritus and insomnia, improved significantly from baseline in both groups (*p* < 0.05), with numerically greater improvements generally observed in the intervention group and more pronounced improvements at day 42 than at day 21.

**Conclusion:**

This study evaluated the incremental value of the emollient under real-world clinical conditions. The soothing and moisturizing emollient cream showed favorable trends in improving skin barrier parameters and clinical symptoms in adults with AD when used as an adjunct to standard therapy. Larger, rigorously designed studies are warranted to confirm its efficacy and long-term clinical value.

## Introduction

Atopic dermatitis (AD) is a common chronic inflammatory skin disease characterized clinically by xerosis, eczematous lesions, and intense pruritus. It tends to recur and can markedly impair quality of life, and its incidence has shown an upward trend in recent years [1]. Numerous studies have demonstrated that impairment of the skin barrier facilitates the penetration of exogenous substances (such as allergens and microorganisms) into the epidermis, triggering inflammatory responses and constituting a key mechanism in the pathogenesis of AD [2–4]. Therefore, repairing and maintaining skin barrier function is one of the fundamental treatment strategies for AD [5]. As basic skin care products, moisturizers and emollients help improve skin barrier function and hydration [6–8].

Research has shown that impairment of skin barrier function in atopic dermatitis is multifactorial, involving diverse mechanisms including, but not limited to, filaggrin deficiency or genetic variation, abnormalities in epidermal lipid organization, and dysbiosis of the cutaneous microbiome. These alterations collectively facilitate the penetration of allergens and pathogenic microorganisms, thereby amplifying cutaneous inflammatory responses [9]. Current clinical guidelines place barrier restoration at the core of basic management for AD, emphasizing that, in addition to anti-inflammatory therapies, the regular use of emollients is essential to reduce transepidermal water loss (TEWL) and to restore the lipid architecture of the stratum corneum [10]. Formulations enriched with physiological lipids, such as ceramides, cholesterol, and fatty acids, have been shown to improve skin hydration, alleviate pruritus, and inhibit *Staphylococcus aureus* colonization, thereby reducing disease recurrence in clinical practice [11]. Furthermore, randomized controlled trials have demonstrated that moisturizers containing multifunctional active ingredients can rapidly enhance epidermal thickness, lipid content, and the expression of key barrier proteins, while concurrently decreasing inflammatory biomarkers, suggesting a potential role in restoring epidermal homeostasis [12]. The present study aims to evaluate the specific effects of the soothing and moisturizing emollient cream on skin barrier parameters in patients with AD, thereby providing scientific evidence for its use as an adjunctive therapeutic option (ChiCTR registration number: ChiCTR2200063420).

## Methods

### Study Design and Ethical Approval

This was a single-center, randomized, double-blind, placebo-controlled, parallel-group clinical trial conducted between October 24, 2022, and October 25, 2024. The study protocol was approved by the Institutional Ethics Committee [Ethics Approval No.: (2022) Lin-shen No. (018)] and was registered with the Chinese Clinical Trial Registry (ChiCTR registration number: ChiCTR2200063420). All participants provided written informed consent prior to enrollment. The completed CONSORT 2025 checklist for this trial is provided as online supplementary material (for all online suppl. material, see https://doi.org/10.1159/spp/aetag011).

### Participants

Adult patients aged 18–65 years with moderate atopic dermatitis (AD) were eligible for inclusion. Moderate AD was defined as a Scoring Atopic Dermatitis (SCORAD) score between 25 and 50, along with the presence of at least one target lesion measuring 1–5 cm in diameter located on the limbs or trunk.

Key exclusion criteria included the presence of skin infection at affected sites; acute AD with erosion or secondary infection; total body surface area involvement of less than 1% or greater than 10%; coexistence of other active inflammatory skin diseases; use of potent topical corticosteroids within 1 week prior to enrollment; and receipt of phototherapy, systemic corticosteroids, or immunosuppressive agents within 4 weeks before study initiation.

A total of 60 eligible patients were enrolled and randomly assigned to the intervention group or the control group in a 1:1 ratio. Two participants in the intervention group and five in the control group discontinued the study. Consequently, 28 (93.3%) participants in the intervention group and 25 (83.3%) participants in the control group completed the trial. Baseline demographic and clinical characteristics were comparable between the two groups, with no statistically significant differences observed (*p* > 0.05) (Table 1). Among the 60 randomized participants, target lesions were located on the lower limbs in 27 (45.0%) participants, the upper limbs in 19 (31.7%) participants, and the trunk in 14 (23.3%) participants.

**Table 1.** Participant information

Variable	Test, NTotal = 30	Control, NTotal = 30	*p* values
Gender, *N* (%)			
*N*	30	30	0.1965
Male	17 (56.7)	12 (40.0)	
Female	13 (43.3)	18 (60.0)	
Age			
N	30	30	0.8616
Mean ± SD	38.97±12.26	39.43±7.91	
Median	37.00	38.50	
Min∼Max	18.00∼64.00	26.00∼59.00	
Q1∼Q3	30.75∼47.00	33.75∼44.50	
Lesion surface area, %			
N	30	30	0.0656
Mean ± SD	5.14±2.27	6.45±2.96	
Median	5.05	6.10	
Min∼Max	1.50∼10.00	1.50∼10.00	
Q1∼Q3	3.50∼6.00	3.38∼9.43	
Target lesion diameter, cm			
*N*	30	30	0.8927
Mean ± SD	3.77±2.29	3.47±1.51	
Median	3.00	3.00	
Min∼Max	1.00∼10.00	1.20∼9.00	
Q1∼Q3	2.00∼4.63	2.23∼4.00	

Data are based on all randomized participants included in the full analysis set.

### Interventions

All participants received standardized background therapy, including oral cetirizine hydrochloride (10 mg once daily in the evening from days 1 to 7), an oral antihistamine commonly used for pruritus control, and topical desonide cream, a low-potency corticosteroid for anti-inflammatory treatment in AD (twice daily from days 1 to 21, followed by application every other evening from days 22 to 42). The target lesion area continued to receive desonide treatment throughout the study period, whereas desonide application to other lesional areas was discontinued once lesion clearance was achieved.

Participants were randomly assigned to receive either the study moisturizer (Weirou Smooth Moisturizing Essence Cream; Guangdong China Resources Shunfeng Pharmaceutical Co., Ltd., Foshan, China), which contained ceramide NP as a key barrier-supporting ingredient, together with squalane, shea butter, sodium hyaluronate, Olea europaea (olive) oil unsaponifiables, and allantoin, or an identical-appearing placebo vehicle, which comprised water, caprylic/capric triglyceride, cetearyl alcohol, dimethicone, cetearyl glucoside, 1,2-hexanediol, sodium carbomer, ammonium acryloyldimethyltaurate/VP copolymer, sucrose polystearate, hydroxyacetophenone, and disodium EDTA. Both products were applied twice daily to the entire body, with a standardized weekly amount of approximately 250 g. Ceramide NP is a phytosphingosine-based ceramide mixture comprising phytosphingosine backbones N-acylated with long-chain fatty acids. The ceramide NP raw material used in the study met a purity specification of ≥95%; however, the exact concentration of ceramide NP in the finished formulation was not disclosed by the manufacturer. Use of non-study moisturizers or emollient products was prohibited throughout the study period.

### Outcome Measures

The skin barrier parameters were changes from baseline in stratum corneum hydration and TEWL measured at the target lesion area and at non-lesional control sites (volar aspect of the upper arm and extensor aspect of the lower leg) on days 21 and 42. Secondary efficacy outcomes included changes from baseline in casual sebum levels and skin pH at the same lesional and non-lesional sites on days 21 and 42, as well as changes in clinical assessment scores, including the Eczema Area and Severity Index (EASI), Scoring Atopic Dermatitis (SCORAD), Dermatology Life Quality Index (DLQI), and visual analogue scale (VAS) scores for pruritus and insomnia.

### Biophysical Measurements

Stratum corneum hydration was measured using the MoistureMeter^®^ SC (Delfin Technologies Ltd., Finland), and TEWL was assessed using the VAPOMETER^®^ (Delfin Technologies Ltd., Finland). Casual sebum levels were measured using the SebumScale^®^ (Delfin Technologies Ltd., Finland). Skin surface pH was measured using a LAQUA pH meter equipped with an ion-sensitive field-effect transistor pH electrode (model 0040N-10D; HORIBA Scientific, Kyoto, Japan).

All biophysical measurements were performed at the target lesion area and non-lesional control sites (volar upper arm and extensor lower leg) at baseline (day 0) and on days 21 and 42. Measurements were conducted under standardized environmental conditions (room temperature 20–22°C; relative humidity 40–60%). Participants rested for at least 20 min prior to assessment. For skin surface pH measurement, the electrode was gently placed in direct contact with the skin at each measurement site, and the pH value was recorded after stabilization of the displayed reading. Three consecutive measurements were obtained for each biophysical parameter at each site, and the mean value was used for analysis.

### Clinical Assessment

Clinical severity of AD was evaluated using the EASI and SCORAD. Quality of life was assessed using the DLQI. Pruritus and sleep disturbance were assessed using VAS scores. All clinical assessments were performed at baseline and on days 21 and 42 by trained dermatologists who were blinded to treatment allocation.

### Sample Size Consideration

No formal a priori sample size calculation was performed for this study. Given the limited availability of eligible participants and the exploratory nature of this randomized controlled trial, a pragmatic sample size of 60 participants was chosen. The study was designed primarily to explore potential trends in skin barrier parameters and clinical outcomes and to generate hypotheses for future confirmatory trials.

### Statistical Analysis

Statistical analyses were conducted using SAS 9.4. Continuous variables were examined for normality and variance homogeneity. Normally distributed continuous variables with equal variances were analyzed using independent-samples *t* tests, whereas normally distributed variables with unequal variances were analyzed using *t* tests with Satterthwaite adjustment. Non-normally distributed continuous variables were analyzed using the Wilcoxon rank-sum test. Categorical variables were evaluated using chi-square or Fisher’s exact tests, and ordinal variables were compared using the Wilcoxon rank-sum test. Primary efficacy outcomes, including changes from baseline in stratum corneum hydration, TEWL, casual sebum levels, and skin surface pH, were analyzed using ANCOVA with baseline values as covariates. All tests were two-sided, and *p* < 0.05 was considered statistically significant.

## Results

### Changes in Skin Barrier Parameters

#### Stratum Corneum Hydration

ANCOVA results showed that stratum corneum hydration levels at the target lesion area, volar upper arm, and extensor lower leg were numerically higher in the experimental group than in the control group on both day 21 and day 42, although the between-group differences did not reach statistical significance. On day 21, significant increases in stratum corneum hydration from baseline were observed at the target lesion and extensor lower leg in the experimental group (*p* < 0.05), whereas in the control group, a significant increase was detected only at the extensor lower leg (*p* < 0.001). By day 42, the experimental group demonstrated a significant increase at the target lesion compared with baseline (*p* < 0.05), whereas the control group continued to show a significant increase at the extensor lower leg (*p* < 0.001). Although no significant between-group differences were observed in changes from baseline at either day 21 or day 42, numerical improvements were generally greater in the experimental group at the target lesion and volar upper arm, while changes at the extensor lower leg were comparable between groups, as shown in Figure 1 and online supplementary Table S1.

**Fig. 1.**
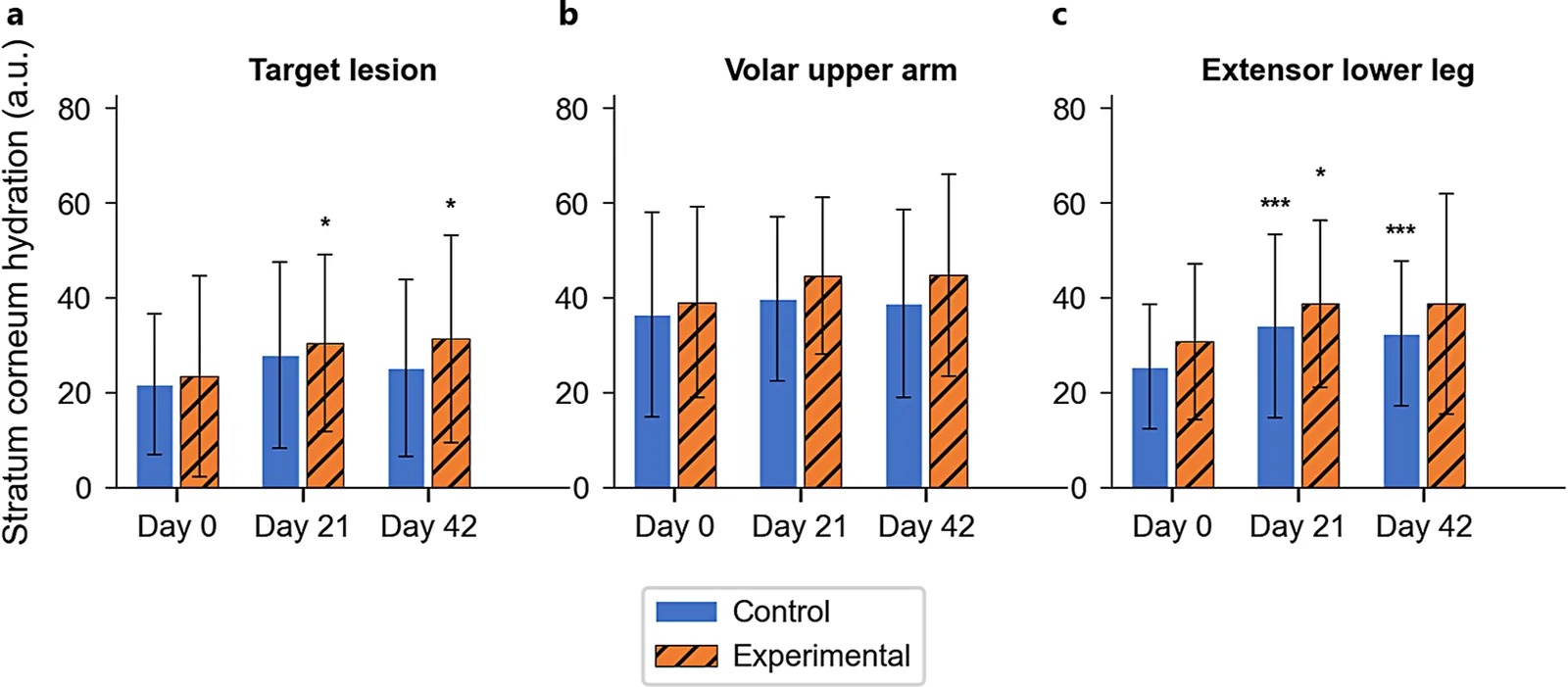
Stratum corneum hydration (a.u.) at baseline, day 21, and day 42. Measurements were obtained at the (**a**) target lesion, (**b**) volar upper arm, and (**c**) extensor lower leg. The experimental group received the ceramide NP-containing moisturizer, whereas the control group received the placebo vehicle; both groups received the same background therapy. Data are presented as mean ± SD, with error bars indicating SD. *p < 0.05, **p < 0.01, and ***p < 0.001 for within-group comparisons with baseline. Exact *p* values are provided in online supplementary Table S1.

#### Transepidermal Water Loss

ANCOVA results indicated that no significant between-group differences in TEWL were observed at the target lesion, volar upper arm, or extensor lower leg on either day 21 or day 42. On day 21, TEWL at the target lesion decreased significantly from baseline in the control group (*p* < 0.01), whereas no significant differences were observed in the experimental group or at the other measurement sites. By day 42, a significant reduction from baseline was observed only at the target lesion in the control group (*p* < 0.01). Numerically, TEWL decreased at the target lesion and volar upper arm in both groups, whereas a slight increase was observed at the extensor lower leg on day 42. No significant between-group differences were found in the changes in TEWL from baseline at either day 21 or day 42, as illustrated in Figure 2 and online supplementary Table S2.

**Fig. 2.**
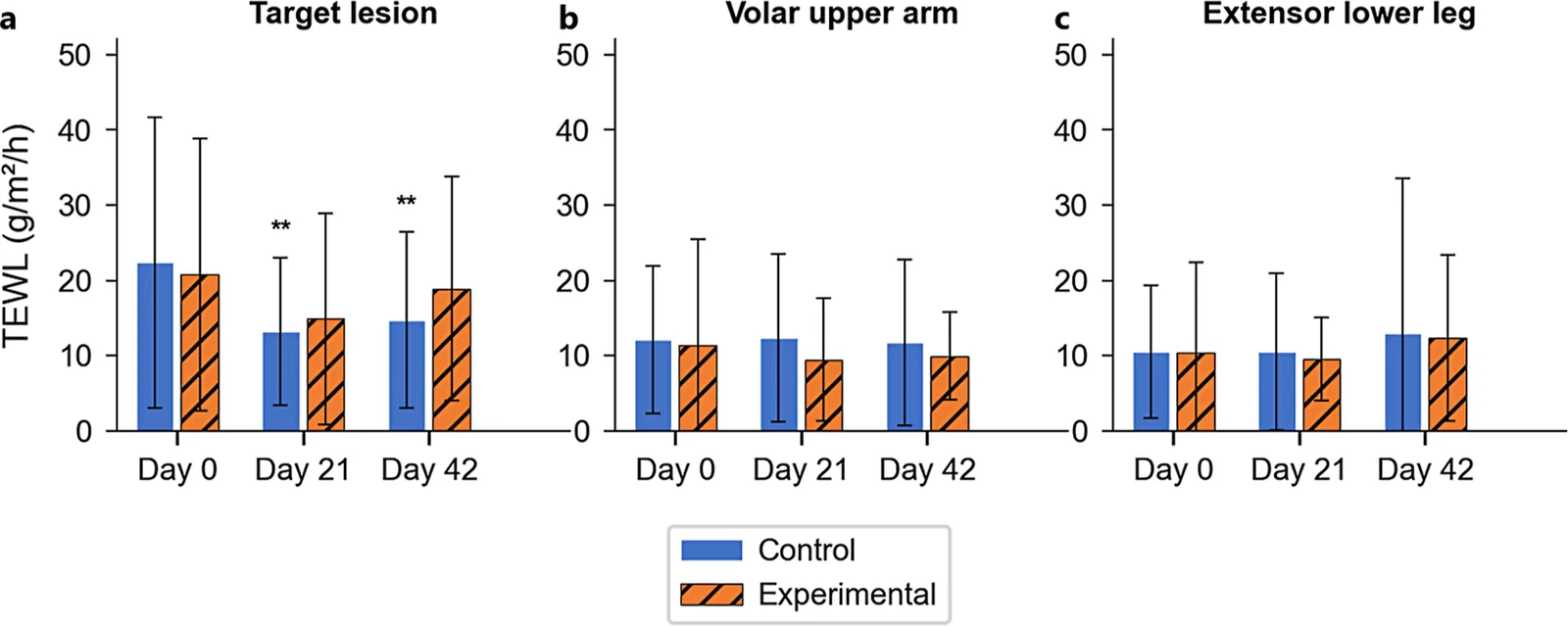
Transepidermal water loss (TEWL; g/m^2^/h) at baseline, day 21, and day 42. Measurements were obtained at the (**a**) target lesion, (**b**) volar upper arm, and (**c**) extensor lower leg. The experimental group received the ceramide NP-containing moisturizer, whereas the control group received the placebo vehicle; both groups received the same background therapy. Data are presented as mean ± SD, with error bars indicating SD. *p < 0.05, **p < 0.01, and ***p < 0.001 for within-group comparisons with baseline. Exact *p* values are provided in online supplementary Table S2.

#### Casual Sebum Levels

ANCOVA results showed that casual sebum levels at all measured sites, including the target lesion, volar upper arm, and extensor lower leg, were numerically higher in the experimental group than in the control group on both day 21 and day 42; however, these between-group differences did not reach statistical significance. Casual sebum levels at all sites in both groups increased significantly from baseline on both day 21 and day 42 (*p* < 0.05). When changes from baseline were compared, no significant between-group differences were observed at either time point, nevertheless, the magnitude of increase was generally greater in the experimental group, as shown in Figure 3 and online supplementary Table S3.

**Fig. 3.**
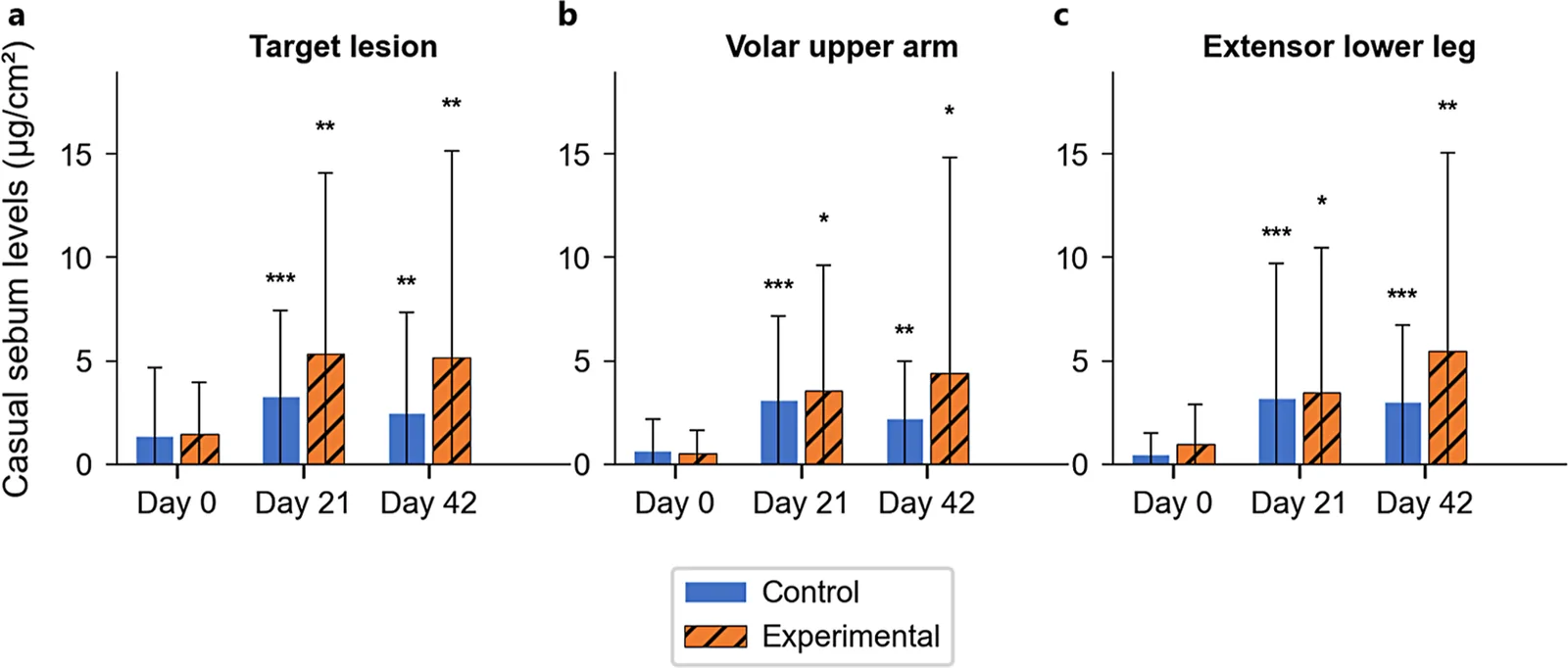
Casual sebum levels (μg/cm^2^) at baseline, day 21, and day 42. Measurements were obtained at the (**a**) target lesion, (**b**) volar upper arm, and (**c**) extensor lower leg. The experimental group received the ceramide NP-containing moisturizer, whereas the control group received the placebo vehicle; both groups received the same background therapy. Data are presented as mean ± SD, with error bars indicating SD. *p < 0.05, **p < 0.01, and ***p < 0.001 for within-group comparisons with baseline. Exact *p* values are provided in online supplementary Table S3.

#### Skin Surface pH

Analysis of skin surface pH demonstrated that there were no statistically significant between-group differences in changes from baseline at either day 21 or day 42. Although a significant increase from baseline was observed at the extensor lower leg in the experimental group at day 42 (*p* = 0.0202), no significant between-group differences were detected at any anatomical site, indicating that the investigational product did not produce a consistent pH-modulating effect across anatomical sites, as shown in Figure 4 and online supplementary Table S4.

**Fig. 4.**
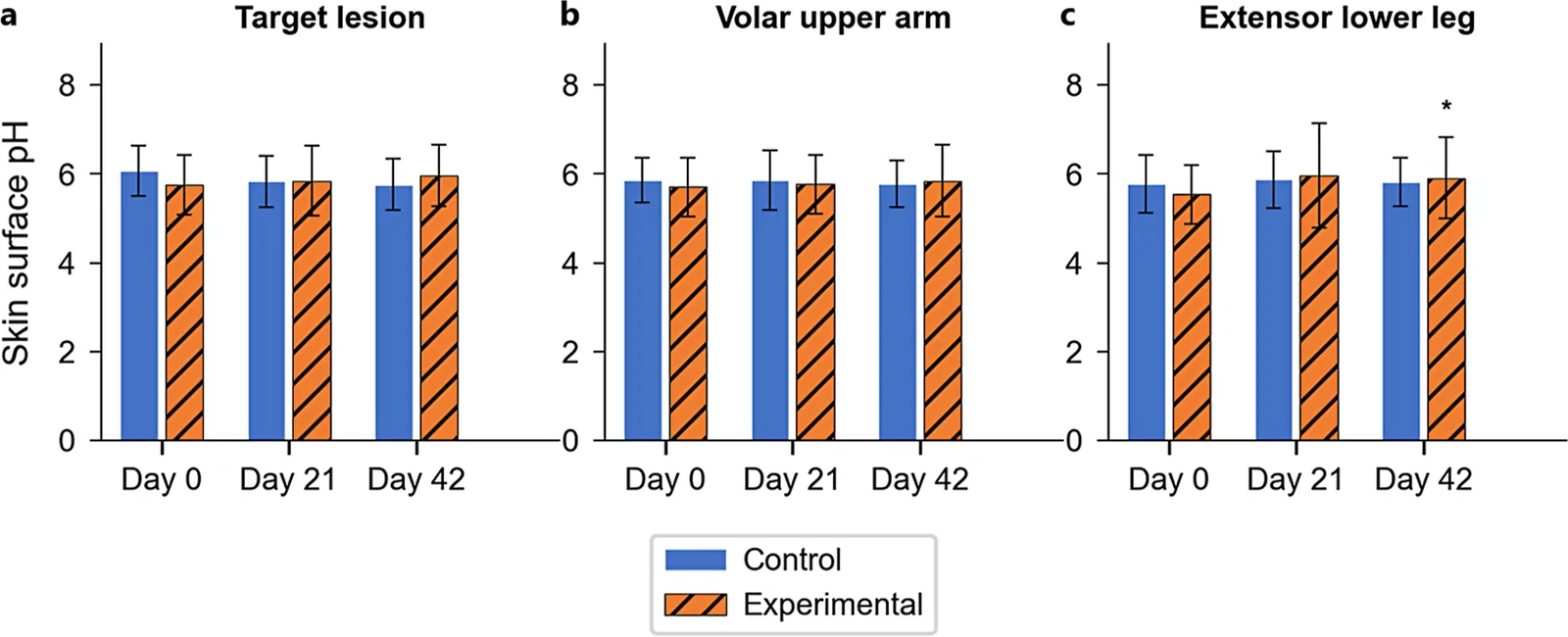
Skin surface pH at baseline, day 21, and day 42. Measurements were obtained at the (**a**) target lesion, (**b**) volar upper arm, and (**c**) extensor lower leg. The experimental group received the ceramide NP-containing moisturizer, whereas the control group received the placebo vehicle; both groups received the same background therapy. Data are presented as mean ± SD, with error bars indicating SD. *p < 0.05, **p < 0.01, and ***p < 0.001 for within-group comparisons with baseline. Exact *p* values are provided in online supplementary Table S4.

### Changes in Clinical Severity and Quality of Life

On days 21 and 42, both the experimental and control groups demonstrated significant improvements from baseline in the EASI, SCORAD, DLQI, and pruritus/insomnia VAS scores (*p* < 0.05). No statistically significant between-group differences were observed in changes from baseline; however, numerically greater improvements were generally observed in the experimental group. Furthermore, in both groups, the improvements observed on day 42 were more pronounced than those observed on day 21, as shown in Figure 5 and online supplementary Table S5.

**Fig. 5.**
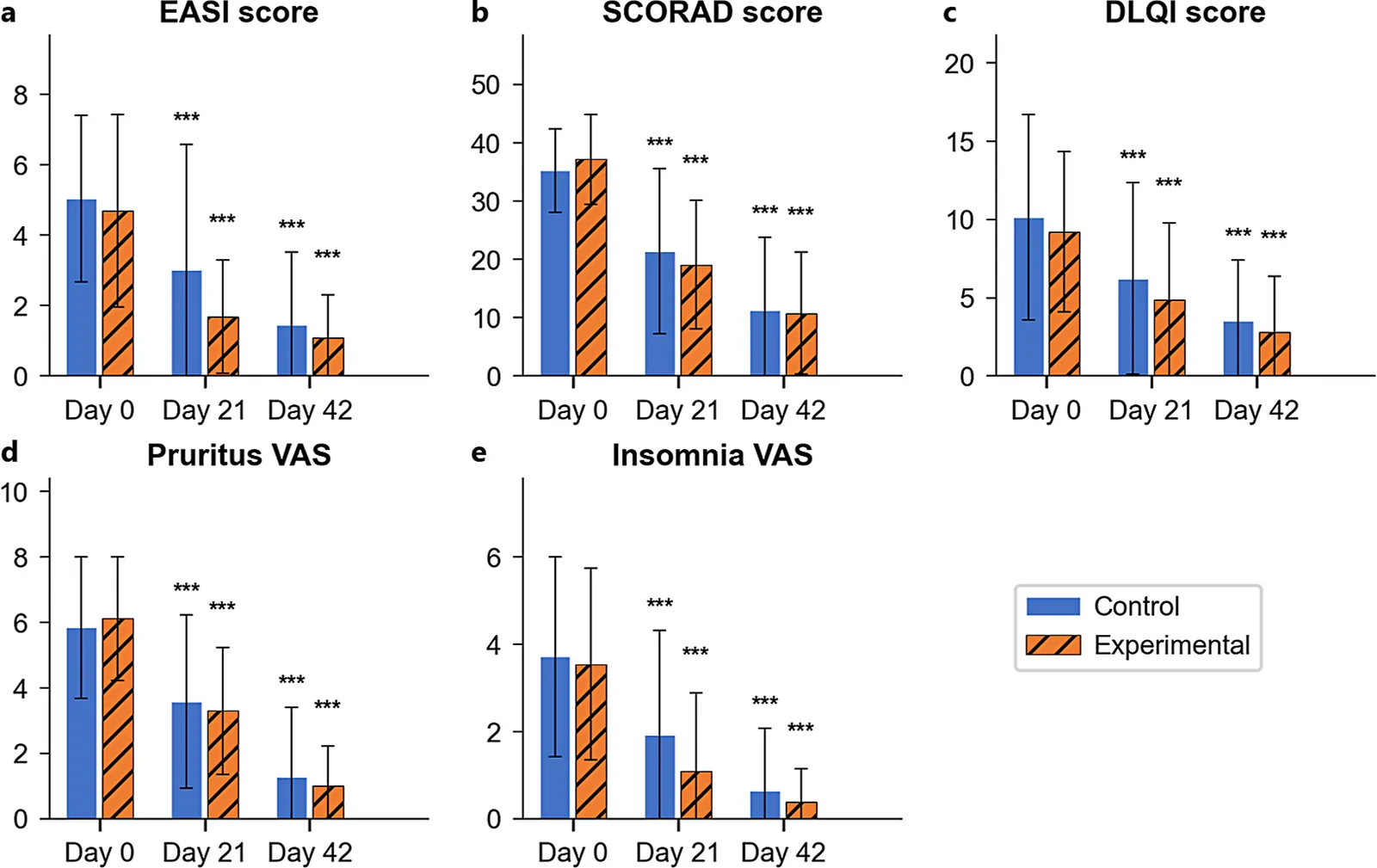
Clinical severity and quality-of-life outcomes at baseline, day 21, and day 42. Outcomes included the (**a**) Eczema Area and Severity Index (EASI), (**b**) SCORing Atopic Dermatitis (SCORAD), (**c**) Dermatology Life Quality Index (DLQI), (**d**) pruritus Visual Analogue Scale (VAS), and (**e**) insomnia VAS scores. The experimental group received the ceramide NP-containing moisturizer, whereas the control group received the placebo vehicle; both groups received the same background therapy. Data are presented as mean ± SD, with error bars indicating SD. *p < 0.05, **p < 0.01, and ***p < 0.001 for within-group comparisons with baseline. Exact *p* values are provided in online supplementary Table S5.

#### Safety and Adverse Events

In this study of 60 participants, one grade 2 adverse event was reported in each of the experimental and control groups, with no significant difference in incidence between groups. All adverse events resolved completely, and no clinically relevant abnormalities were observed in vital signs, physical examinations, or other safety assessments. Overall, the investigational product demonstrated a favorable safety and tolerability profile.

## Discussion

AD has a complex pathogenesis, with skin barrier dysfunction recognized as its most critical pathological feature. This dysfunction is typically characterized by increased TEWL, reduced stratum corneum hydration, elevated skin pH, and decreased sebum content [13, 14]. The use of emollients is universally endorsed as first-line foundational therapy for AD in both national and international clinical practice guidelines [15, 16], as these agents help prevent water loss, restore skin barrier integrity, and support overall skin homeostasis.

The study moisturizer contains ceramide NP, which is intended to support restoration of the stratum corneum lipid barrier. Previous studies have reported barrier-related benefits of ceramide-based formulations. Choi et al. [17] demonstrated enhanced recovery of experimentally disrupted human skin following application of a plant-oil-derived ceramide NP cream. In 40 patients with mild-to-moderate AD, a pseudoceramide moisturizer increased median stratum corneum hydration and reduced objective SCORAD by approximately 24% after 4 weeks, although TEWL did not change significantly [18]. In 100 adults with moderate eczema, a ceramide-dominant regimen reduced EASI by approximately 45% after 28 days and produced greater improvements in hydration and TEWL than placebo [19]. In the present study, stratum corneum hydration at the target lesion in the experimental group increased from 23.54 to 31.39 a.u. at day 42, corresponding to an increase of approximately 33.3%, whereas TEWL numerically decreased from 20.78 to 18.91 g/m^2^/h, corresponding to a reduction of approximately 9.0%. However, the between-group differences were not statistically significant. These findings are directionally consistent with previous reports and support a potential contribution of ceramide NP-containing formulations to skin barrier improvement in AD, although direct comparisons should be interpreted cautiously because of differences in study design, population, concomitant treatment, and product composition.

It is also noteworthy that the upper arm and extensor lower leg differ substantially in their baseline physiological characteristics, as hydration and barrier-related parameters vary considerably across anatomical sites [20]. Although improvements were observed at both anatomical sites in the present study, the trial was not designed to formally compare treatment responses between different anatomical locations. Furthermore, although TEWL is widely used as an indicator of skin barrier integrity, it is influenced by multiple physiological and environmental factors, particularly anatomical site and ambient conditions [21]. Regional differences in skin structure, lipid composition, baseline hydration, and environmental exposure may contribute to greater measurement variability at the extensor lower leg and may partly account for the numerical increase in TEWL observed at this site at day 42. However, neither the within-group changes nor the between-group difference reached statistical significance. Given the similar direction of change in both groups and the observed measurement variability, this site-specific finding should be interpreted cautiously and considered alongside TEWL findings at the other anatomical sites and changes in other barrier-related parameters.

Surface sebum measurements reflect sebaceous lipids present at the skin surface and may be altered in patients with atopic dermatitis (AD). However, they do not directly quantify the intercellular lipid matrix of the stratum corneum, which is primarily composed of ceramides, cholesterol, and free fatty acids [22]. In this study, casual sebum levels at the target lesion sites, as well as at the volar upper arm and extensor lower leg, were similarly low in both groups at baseline. Following treatment, increases in casual sebum levels were observed in both groups, with a greater magnitude of increase in the experimental group. These findings suggest that the ceramide NP-containing emollient may favorably influence skin surface lipid availability. Together with improvements observed in other barrier-related parameters, these findings support the potential role of ceramide NP-containing emollients in promoting epidermal barrier recovery, potentially through mechanisms involving restoration of stratum corneum lipid organization.

Normal skin typically exhibits a mildly acidic pH of approximately 5.0; however, barrier dysfunction in AD often leads to a more alkaline skin surface, with an average pH of approximately 5.7 and values reaching up to 7.3 in severe cases [23, 24]. In the present study, no statistically significant changes in skin surface pH were observed at the target lesion or volar upper arm in either group. Although a significant increase was observed at the extensor lower leg in the experimental group at day 42, no significant between-group differences were detected at any anatomical site. Therefore, the investigational product did not demonstrate a consistent pH-modulating effect under the conditions of this study. The study moisturizer contains ceramide NP and other moisturizing ingredients that are primarily intended to support lipid-barrier restoration and hydration rather than to acidify the skin surface. As skin surface pH is regulated by multiple factors, such as natural moisturizing factor metabolism and acidic secretions including lactic acid and free amino acids [25], normalization of AD-associated alkalinity may require formulations that combine barrier-repair agents with pH-modulating or mildly acidic components [26, 27]. The absence of a specific pH-adjusting system in the tested formulation may partly explain the lack of consistent pH changes across anatomical sites in this study.

With respect to clinical outcomes, compared with the control group, the experimental group exhibited numerically greater improvements from baseline across all measures – including DLQI, EASI, pruritus/insomnia VAS, and SCORAD scores – with these benefits progressing further at day 42 compared to day 21. These findings suggest that restoration of the skin barrier may contribute to the alleviation of skin lesions and lichenification in patients with AD, thereby improving quality of life. No serious adverse events were reported among the 60 participants enrolled in this study, and all adverse events resolved completely, indicating that the product was generally safe and well tolerated for clinical use.

In this study, an important consideration in interpreting these findings is the use of potent background therapy in both groups. All participants received oral cetirizine and topical desonide, a low-potency topical corticosteroid with well-established efficacy in atopic dermatitis [28]. While this treatment layering reflects routine clinical practice, it may have produced a ceiling effect, limiting the detection of modest between-group differences attributable to the adjunctive emollient. Notably, this study was designed to assess the incremental value of the emollient under real-world conditions rather than in a corticosteroid-free setting. In this context, the consistent trend toward greater improvement in barrier-related parameters in the intervention group suggests a potential adjunctive benefit that warrants confirmation in larger studies with longer follow-up focused on maintenance therapy and relapse prevention.

### Limitations

Several limitations should be noted. Despite the randomized, double-blind design, this single-center study had a relatively small sample size and no formal a priori sample size calculation, which may have limited the statistical power to detect between-group differences. Based on the observed effect sizes, a substantially larger sample would be required in future studies. Accordingly, the findings should be interpreted as exploratory and hypothesis-generating. In addition, the short follow-up period precluded assessment of long-term outcomes, and individual variability as well as reliance on some subjective clinical measures may have introduced variability despite blinding. The lack of early assessment time points also prevented evaluation of rapid initial changes that may occur shortly after moisturizer application, particularly for hydration-related parameters. Furthermore, outdoor environmental conditions (e.g., ambient temperature and humidity) were not prospectively recorded during the study period. Although all assessments were performed under standardized indoor conditions and randomization would be expected to minimize imbalance in environmental exposure between groups, the potential influence of these factors on skin barrier measurements cannot be completely excluded. Larger, multicenter studies with longer follow-up are therefore warranted.

## Conclusion

In this randomized, double-blind, placebo-controlled trial, adjunctive use of the soothing and moisturizing emollient cream was associated with improvements in skin barrier parameters and clinical outcomes in adults with moderate atopic dermatitis. Although the intervention group showed more favorable trends than the placebo group across several endpoints, the between-group differences in changes from baseline did not reach statistical significance. Therefore, these findings should be interpreted with caution and are best considered hypothesis-generating. Larger, multicenter studies with longer follow-up are warranted to clarify the potential clinical benefits and long-term value of ceramide NP-containing emollients in atopic dermatitis.

## Acknowledgments

The authors gratefully acknowledge the dedication and cooperation of all study participants. We also extend our sincere thanks to the clinical staff at the Department of Dermatology, Huashan Hospital, Fudan University, for their invaluable support in patient care and data collection throughout the trial.

## Statement of Ethics

This study protocol was reviewed and approved by the Institutional Review Board of Huashan Hospital, Fudan University, Shanghai, China, Approval No. (2022) Lin-shen No. (018). Written informed consent was obtained from all individual participants included in the study prior to any study-related procedures.

## Conflict of Interest Statement

The authors have no conflicts of interest to declare.

## Funding Sources

This study was not supported by any sponsor or funder.

## Author Contributions

Conceptualization: L.-H.X. Methodology: L.-H.X., C.-Y.G., and J.-Y.Y. Investigation: C.-Y.G., J.-Y.Y., H.-B.Y., and C.-Y.H. Data curation, writing – original draft, and formal analysis: C.-Y.G. and J.-Y.Y. Writing – review and editing: H.-B.Y., C.-Y.H., and L.-H.X. Supervision and project administration: L.-H.X. C.-Y.G. and J.-Y.Y. contributed equally to this work and are co-first authors.

## Data Availability

All data generated or analyzed during this study are included in this article and its online supplementary material files. Due to the sensitive nature of the clinical data involving human participants, the raw datasets are not publicly available to protect participant privacy. Further inquiries regarding conditional data access for the purposes of verification or research collaboration can be directed to the corresponding author upon reasonable request, subject to the approval of the original Ethics Committee.
